# Supplementary material for: CCN2 Aggravates the Immediate Oxidative Stress–DNA Damage Response following Renal Ischemia–Reperfusion Injury
Source: Antioxidants (Basel). 2021 Dec 20;10(12):2020. doi: 10.3390/antiox10122020 (PMC8698829; doi:10.3390/antiox10122020)
Supplement: Supplementary file 1 [file antioxidants-10-02020-s001.zip › Figure S1.pptx]

## Slide 1
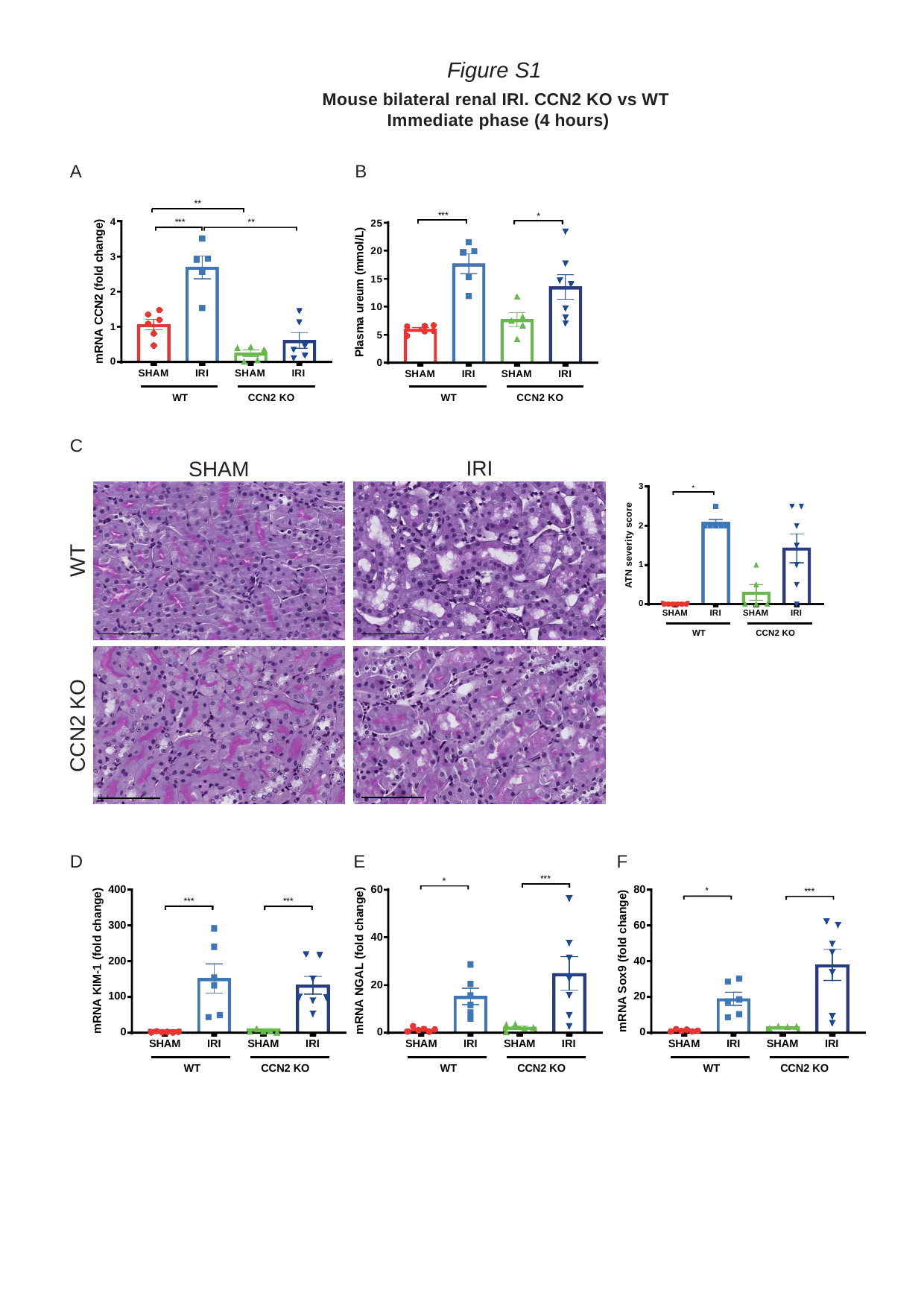

Figure S1
Mouse bilateral renal IRI. CCN2 KO vs WT Immediate phase (4 hours)
A
B
**
***
*
4
**
***
25
mRNA CCN2 (fold change)
Plasma ureum (mmol/L)
20
3
15
2
10
1
5
0
0
SHAM
IRI
SHAM
IRI
SHAM
IRI
SHAM
IRI
WT
CCN2 KO
WT
CCN2 KO
C
IRI
SHAM
3
*
ATN severity score
2
WT
1
0
SHAM
IRI
SHAM
IRI
WT
CCN2 KO
CCN2 KO
D
E
F
***
*
60
400
80
*
***
mRNA NGAL (fold change)
mRNA KIM-1 (fold change)
mRNA Sox9 (fold change)
***
***
300
60
40
200
40
20
100
20
0
0
0
SHAM
IRI
SHAM
IRI
SHAM
IRI
SHAM
IRI
SHAM
IRI
SHAM
IRI
WT
CCN2 KO
WT
CCN2 KO
WT
CCN2 KO
