# Supplementary material for: CCN2 Aggravates the Immediate Oxidative Stress–DNA Damage Response following Renal Ischemia–Reperfusion Injury
Source: Antioxidants (Basel). 2021 Dec 20;10(12):2020. doi: 10.3390/antiox10122020 (PMC8698829; doi:10.3390/antiox10122020)
Supplement: Supplementary file 1 [file antioxidants-10-02020-s001.zip › Figure S2.pptx]

## Slide 1
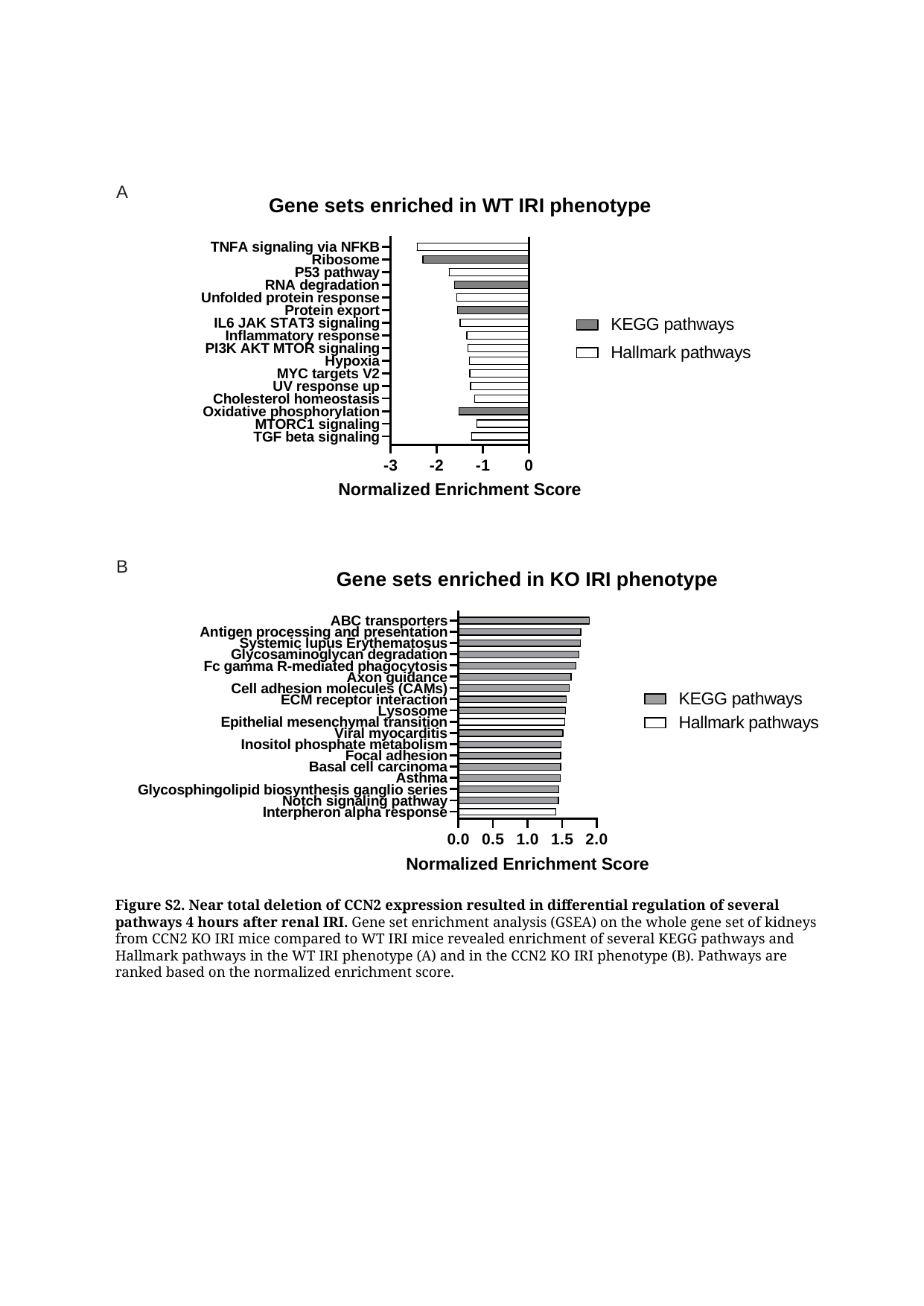

A
B
Figure S2. Near total deletion of CCN2 expression resulted in differential regulation of several pathways 4 hours after renal IRI. Gene set enrichment analysis (GSEA) on the whole gene set of kidneys from CCN2 KO IRI mice compared to WT IRI mice revealed enrichment of several KEGG pathways and Hallmark pathways in the WT IRI phenotype (A) and in the CCN2 KO IRI phenotype (B). Pathways are ranked based on the normalized enrichment score.
