# Supplementary material for: CCN2 Aggravates the Immediate Oxidative Stress–DNA Damage Response following Renal Ischemia–Reperfusion Injury
Source: Antioxidants (Basel). 2021 Dec 20;10(12):2020. doi: 10.3390/antiox10122020 (PMC8698829; doi:10.3390/antiox10122020)
Supplement: Supplementary file 1 [file antioxidants-10-02020-s001.zip › Figure S3.pptx]

## Slide 1
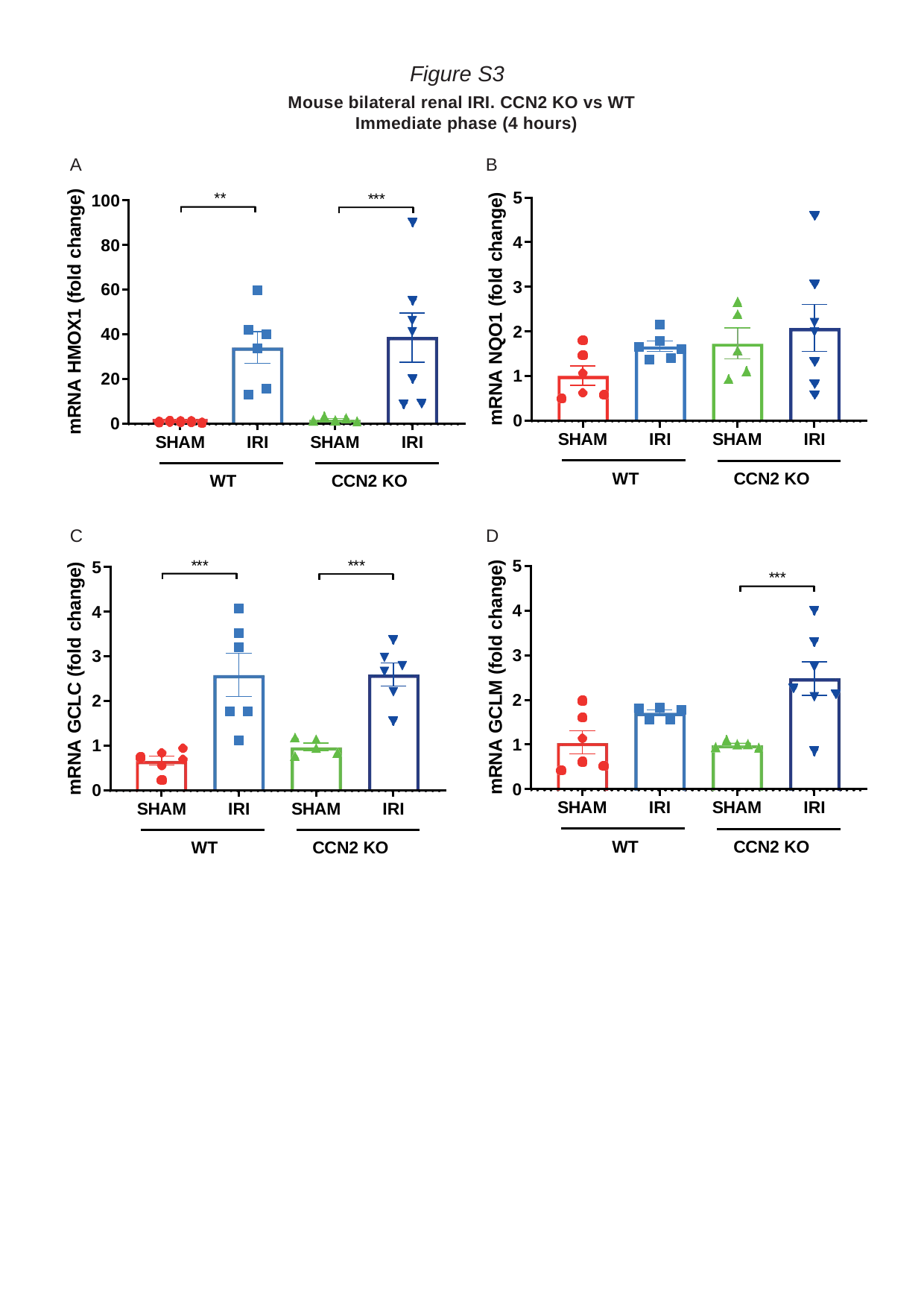

Figure S3
Mouse bilateral renal IRI. CCN2 KO vs WT Immediate phase (4 hours)
A
B
5
**
***
mRNA HMOX1 (fold change)
100
mRNA NQO1 (fold change)
4
80
3
60
2
40
1
20
0
0
SHAM
IRI
SHAM
IRI
SHAM
IRI
SHAM
IRI
WT
CCN2 KO
WT
CCN2 KO
C
D
5
***
***
5
mRNA GCLM (fold change)
mRNA GCLC (fold change)
***
4
4
3
3
2
2
1
1
0
0
SHAM
IRI
SHAM
IRI
SHAM
IRI
SHAM
IRI
WT
CCN2 KO
WT
CCN2 KO
