# Supplementary material for: CCN2 Aggravates the Immediate Oxidative Stress–DNA Damage Response following Renal Ischemia–Reperfusion Injury
Source: Antioxidants (Basel). 2021 Dec 20;10(12):2020. doi: 10.3390/antiox10122020 (PMC8698829; doi:10.3390/antiox10122020)
Supplement: Supplementary file 1 [file antioxidants-10-02020-s001.zip › Table S1. qPCR primers.pdf]

Table S1. Primers used for real-time polymerase chain reaction

Gene	SYBR Green primer sequence or Taqman Gene Expression Assay	accession	amplicon size
HMOX1	Fwd: AGGGTCAGGTGTCCAGAGAA	NM_010442.2	72
	Rev: CTTCCAGGGCCGTGTAGATA		
NQO1	Fwd: AGCGTTCGGTATTACGATCC	NM_008706.5	68
	Rev: AGTACAATCAGGGCTCTTCTCG		
GCLC	Fwd: AGATGATAGAACACGGGAGGAG	NM_010295.2	62
	Rev: TGATCCTAAAGCGATTGTTCTTC		
GCLM	Fwd: TGACTCACAATGACCCGAAA	NM_008129.4	79
	Rev: TCAATGTCAGGGATGCTTTCT		
HMGB1A	Fwd: TGGGCGACTCTGTGCCTC	NM_010439.4	72
	Rev: GCCTCTCGGCTTTTTAGGATC		
TBP	Mm00446971_m1	NM_013684.3	93
CCN2	Mm01192932_g1	NM_010217.2	61
KIM1	Mm00506686_m1	NM_134248.2	94
NGAL	Mm01324470_m1	NM_008491.1	84
SOX9	Mm00448840_m1	NM_011448.4	101
CDKN1A	Mm00432448_m1	NM_007669.4	96
GADD45A	Mm00432802_m1	NM_007836.1	59

Fwd = forward primer; Rev = reverse primer.
