# Supplementary material for: CCN2 Aggravates the Immediate Oxidative Stress–DNA Damage Response following Renal Ischemia–Reperfusion Injury
Source: Antioxidants (Basel). 2021 Dec 20;10(12):2020. doi: 10.3390/antiox10122020 (PMC8698829; doi:10.3390/antiox10122020)
Supplement: Supplementary file 1 [file antioxidants-10-02020-s001.zip › Table S3. Top DEGs WT IR vs KO IRI.pdf]

Table S3. Differentially expressed genes with the largest fold down- or upregulation in the pairwise comparison between CCN2 KO IRI mice and WT IRI mice.

Gene	Full gene name	log2 FC §	lfcSE	p value &
Rap2b*	RAP2B (member of RAS oncogene family)	2,106	0,238	0,000
Frat2*	FRAT regulator of WNT signaling pathway 2	1,902	0,297	0,000
Ier2*	immediate early response 2	1,780	0,209	0,000
Cxcl1	C-X-C motif chemokine ligand 1	1,718	0,306	0,000
Cebpb	CCAAT enhancer binding protein beta	1,686	0,215	0,000
Cxcl10	C-X-C motif chemokine ligand 10	1,667	0,200	0,000
Kif23	kinesin family member 23	1,660	0,237	0,000
Csrnp1	cysteine and serine rich nuclear protein 1	1,505	0,227	0,000
Vgf	VGF nerve growth factor inducible	1,486	0,313	0,000
Maff	MAF bZIP transcription factor F	1,484	0,307	0,000
Junb	jun B proto-oncogene	1,482	0,186	0,000
Btg1	BTG anti-proliferation factor 1	1,478	0,190	0,000
Pde10a	phosphodiesterase 10A	1,449	0,268	0,000
Ppp1r15a	protein phosphatase 1 regulatory subunit 15A	1,427	0,279	0,000
Btg2	BTG anti-proliferation factor 2	1,376	0,210	0,000
Klhdc7a	kelch domain containing 7A	-1,681	0,212	0,000
Flrt1	fibronectin leucine rich transmembrane protein 1	-1,665	0,231	0,000
Fads6	fatty acid desaturase 6	-1,644	0,227	0,000
Il22ra1	interleukin 22 receptor subunit alpha 1	-1,612	0,313	0,000
Ccnd1	cyclin D1	-1,526	0,182	0,000
Prox1	prospero homeobox 1	-1,500	0,303	0,000
Gpam	glycerol-3-phosphate acyltransferase (mitochondrial)	-1,485	0,159	0,000
Tmem88b	transmembrane protein 88B	-1,482	0,246	0,000
Adamts15	ADAM metallopeptidase with thrombospondin type 1 motif 15	-1,468	0,248	0,000
Syt17	synaptotagmin 17	-1,462	0,251	0,000
Cyp2u1	cytochrome P450 family 2 subfamily U member 1	-1,455	0,279	0,000
Bsnd	barttin CLCNK type accessory beta subunit	-1,449	0,186	0,000
Fam78a	family with sequence similarity 78 member A	-1,447	0,272	0,000
Cyp26b1	cytochrome P450 family 26 subfamily B member 1	-1,408	0,299	0,000
Tnrc18	trinucleotide repeat containing 18	-1,399	0,210	0,000

§ Positive Log2 fold change values represent lower expression in CCN2 KO IRI vs WT IRI; Negative Log2 fold change values represent higher expression in CCN2 KO IRI vs WT IRI
& False discovery rate adjusted p-values
Abbreviations: CCN2 = cellular communication network factor 2; KO = knockout; IRI = ischemia-reperfusion injury; WT = wild type; FC = fold change; SE = standard error
